# Mechanical sensitization of cutaneous sensory fibers in the spared nerve injury mouse model

**DOI:** 10.1186/1744-8069-9-61

**Published:** 2013-11-29

**Authors:** Amanda K Smith, Crystal L O’Hara, Cheryl L Stucky

**Affiliations:** 1 Department of Cell Biology, Neurobiology, and Anatomy, Medical College of Wisconsin, 8701 Watertown Plank Road, Milwaukee, WI, USA

**Keywords:** Neuropathic, Nociceptor, Sensory neuron, C fiber, A fiber, Hyperalgesia, Mechanotransduction

## Abstract

**Background:**

The spared nerve injury (SNI) model of neuropathic pain produces robust and reproducible behavioral mechanical hypersensitivity. Although this rodent model of neuropathic pain has been well established and widely used, peripheral mechanisms underlying this phenotype remain incompletely understood. Here we investigated the role of cutaneous sensory fibers in the maintenance of mechanical hyperalgesia in mice post-SNI.

**Findings:**

SNI produced robust, long-lasting behavioral mechanical hypersensitivity compared to sham and naïve controls beginning by post-operative day (POD) 1 and continuing through at least POD 180. We performed teased fiber recordings on single cutaneous fibers from the spared sural nerve using *ex vivo* skin-nerve preparations. Recordings were made between POD 16–42 after SNI or sham surgery. Aδ-mechanoreceptors (AM) and C fibers, many of which are nociceptors, from SNI mice fired significantly more action potentials in response to suprathreshold mechanical stimulation than did fibers from either sham or naïve control mice. However, there was no increase in spontaneous activity.

**Conclusions:**

To our knowledge, this is the first study evaluating the contribution of primary afferent fibers in the SNI model. These data suggest that enhanced suprathreshold firing in AM and C fibers may play a role in the marked, persistent mechanical hypersensitivity observed in this model. These results may provide insight into mechanisms underlying neuropathic pain in humans.

## Findings

### Background

Peripheral neuropathic pain results from complete or partial lesion to peripheral nerves [1,2]. Occurring in many neurological disorders, neuropathic pain affects 6-8% of the population and is characterized by spontaneous and stimulus-evoked pain [2]. The mechanisms driving nerve injury-induced hyperalgesia are not well understood making treatments sub-optimal [1-4]. Several rodent models of neuropathic pain have been developed including, chronic constriction injury (CCI) [5], partial sciatic nerve injury [6], and spinal nerve ligation (SNL) [7]. These models often involve variability within cohorts [1,4] and present a challenge in determining the role of injured versus non-injured, intact sensory afferents in neuropathic pain because they involve a high degree of co-mingling of intact and injured axons distal to the lesion [8].

We used the spared nerve injury (SNI) model of neuropathic pain which produces a pronounced, long-lasting and reproducible behavioral phenotype characterized by intense mechanical allodynia and hyperalgesia that mimics many features of clinical neuropathic pain [1,3,9]. SNI comprises complete transection of two of the three sciatic nerve branches (tibial and common peroneal), leaving the sural nerve intact [1]. Furthermore, SNI involves minimal co-mingling of intact and injured axons distal to the lesion [1], thereby allowing investigators to specifically target non-directly-injured nerve fibers. Although SNI is well established, the peripheral mechanisms contributing to the pain phenotype are not clear. While teased fiber recordings have been used to investigate peripheral sensitization in SNL in monkey and rat [10,11], and in CCI in rat [12,13], to our knowledge, these experiments have not been performed in the SNI model. Thus, the goal of this study was to determine whether intact cutaneous afferent fibers from the spared sural nerve are sensitized to mechanical stimuli and thereby, may contribute to the maintenance of mechanical hypersensitivity after SNI.

### SNI mice exhibit long-lasting behavioral mechanical hypersensitivity

**Figure 1:**
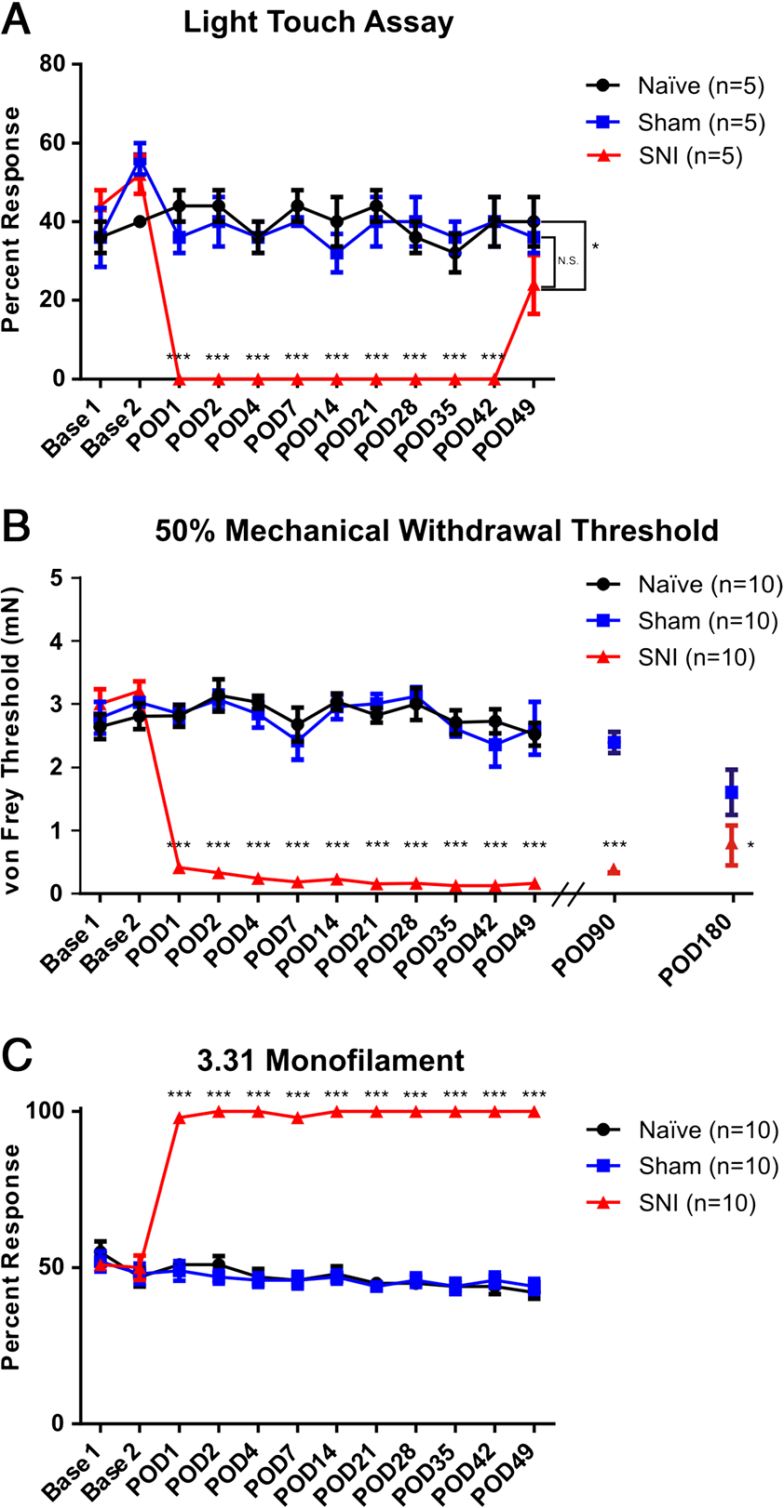
**SNI mice exhibit prominent behavioral hypersensitivity. A)** In response to dynamic stroke of a puffed cotton swab, SNI mice show significant desensitization of the tibial territory beginning POD 1 and continuing through POD 42 compared to sham or naïve animals (***p<0.005). At POD 49 SNI mice begin to regain sensation in the tibial territory, in that SNI mice still showed some sensitization compared to naïve animals (*p<0.05) but not sham animals (p>0.05). Treatments were compared across time using a repeated measure 2-way ANOVA with Tukey’s *post hoc* comparisons. **B)** SNI mice show a significant decrease in the 50% mechanical withdrawal threshold beginning POD 1 and continuing through POD 49 compared to sham or naïve mice (***p<0.005). Furthermore, SNI mice continue to show a significant decrease in mechanical withdrawal threshold at POD 90 compared to sham (***p<0.005) and at POD 180 compared to sham (*p<0.05). Treatments were compared across time using a 2-way ANOVA with Bonferroni *post hoc* tests. SNI and sham treatments were compared at POD 90 and POD 180 using Mann–Whitney U tests. **C)** In response to repeated stimulus with a 3.31 mN monofilament, SNI mice exhibit prominent hypersensitivity beginning POD 1 and continuing through at least POD 49 compared to sham or naïve animals (***p<0.005). Treatments were compared across time using a 2 way-ANOVA with Bonferroni *post hoc* tests. Error bars for all three graphs indicate S.E.M.

As previously reported, SNI mice exhibited pronounced hypersensitivity to mechanical stimuli compared to sham and naïve animals beginning by post-operative day (POD) 1 and continuing for at least 6 months post-surgery (Figure 1). The dynamic component of the Light Touch Behavioral Assay [14] was used as a control to ensure adequate denervation of the tibial territory post-SNI injury. SNI mice showed significant tibial desensitization, measured by percent response to a puffed cotton swab applied to the tibial territory of the glabrous skin, from POD 1–42 (Figure 1A, p < 0.005), which was expected because of transection of the tibial nerve and subsequent denervation of the skin territory. However, by POD 49, sensation in the tibial area began to return (Figure 1A). In sural nerve-targeted behavioral testing, SNI mice showed a significant decrease in paw withdrawal threshold by POD 1 through POD 180 (Figure 1B, p < 0.005) and exhibited a significantly higher percent response to the suprathreshold 3.31 mN monofilament from POD 1–49 (Figure 1C, p < 0.005). Locomotor activity of the mice did not differ between groups (data not shown). Complete transection of the tibial and common peroneal nerves was validated post-mortem. Overall, these results parallel those found in rat [1] and previously shown in mouse [3,9].

### Aδ and C fibers from SNI mice exhibit enhanced mechanical firing

**Figure 2:**
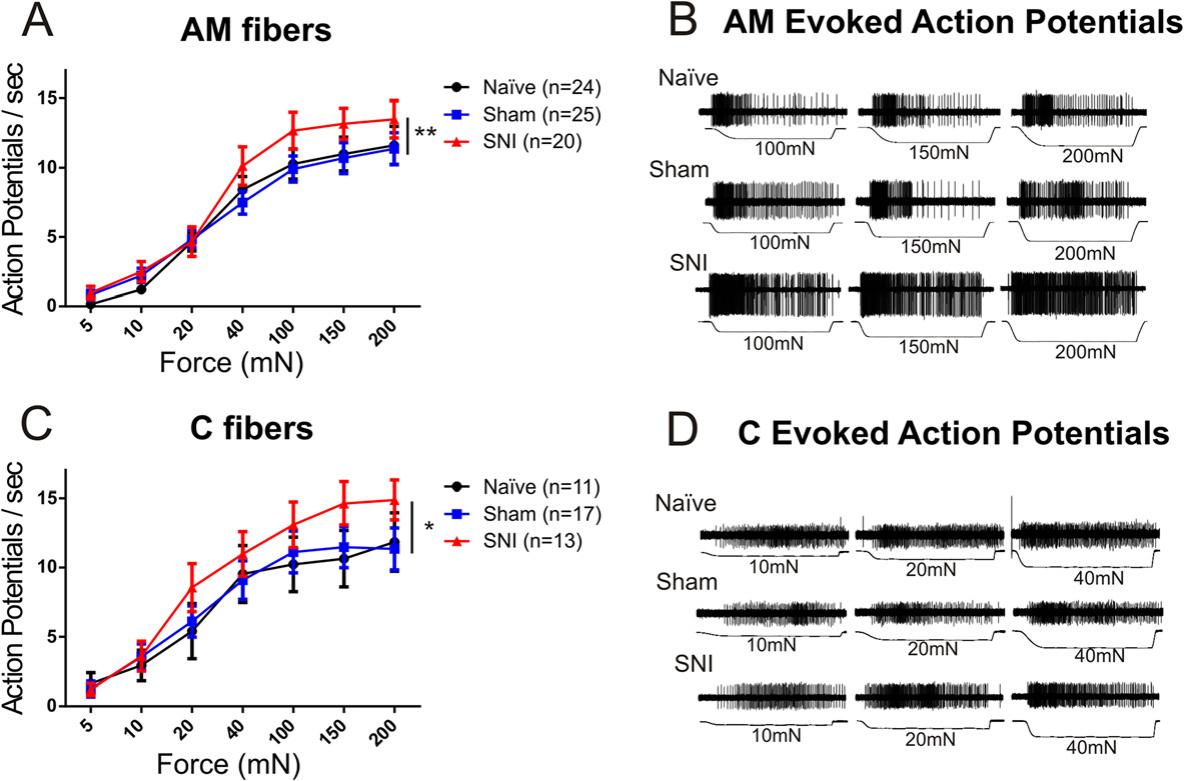
**Aδ-Mechanoreceptor and C fibers in SNI mice exhibit enhanced mechanical firing.** Using *ex vivo* skin nerve recordings, 10 sec mechanical stimuli ranging 5-200 mN were applied to the receptive field of each fiber using a 0.8 mm probe. All recordings were performed on the sural nerve and its innervating territory. **A)** Aδ-Mechanoreceptor (AM) fibers fired an average 22% more action potentials per second across all forces compared to sham or to naïve animals (**p<0.01). **B)** Examples of AM fiber action potentials evoked by sustained mechanical stimuli in naïve, sham and SNI treatment groups. **C)** C fibers fired an average 24% more action potentials per second across all forces compared to sham and 28% more compared to naïve animals (*p<0.05). **D)** Examples of C fiber action potentials evoked by sustained mechanical stimuli in naïve, sham and SNI treatment groups. Number of action potentials fired per second was compared across all forces and treatment groups using a 2-way ANOVA with Bonferroni *post hoc* comparisons. Error bars indicate S.E.M.

**Figure 3:**
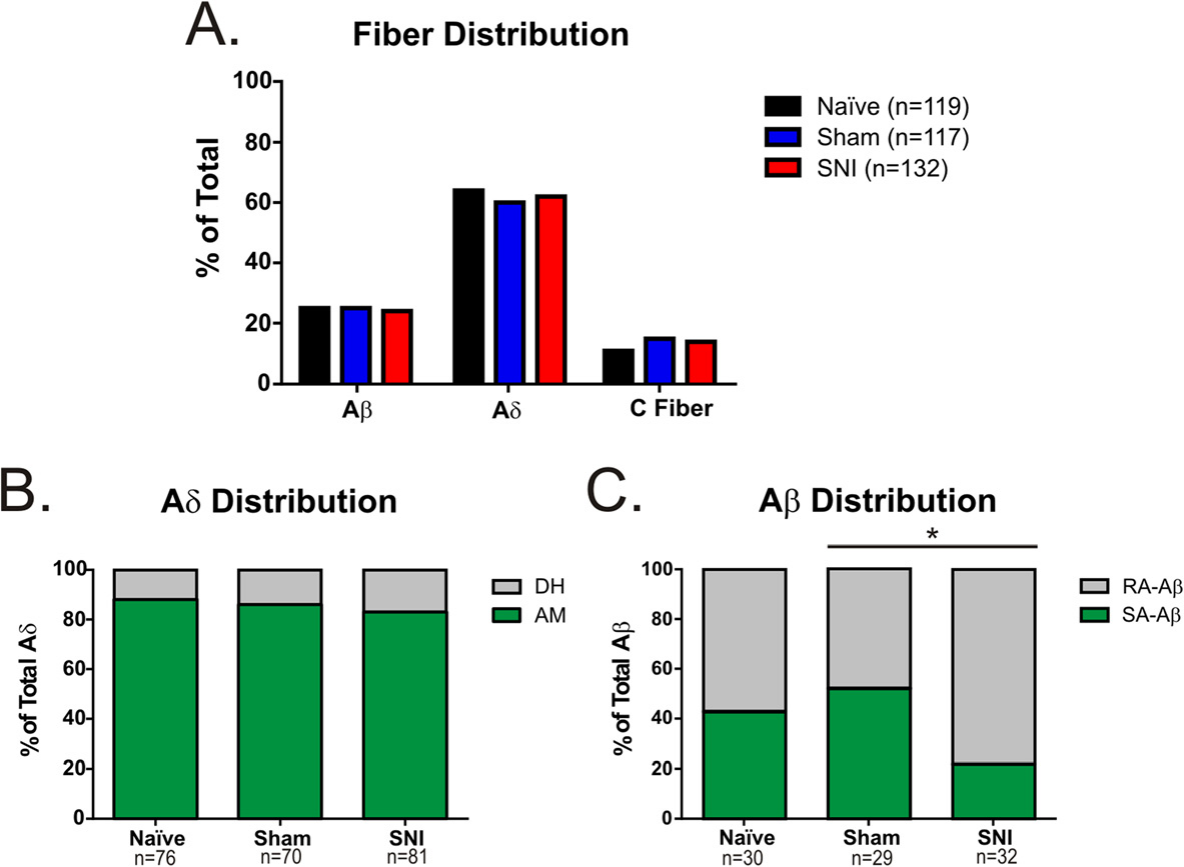
**Distribution of cutaneous fiber types in SNI mice.** Since SNI mice fired significantly more action potentials per second across all forces, we compared the percent distribution of each fiber type across treatment groups. **A)** There was not a significant difference in percent fiber distribution in SNI mice compared to sham or naïve animals (p>0.05). **B)** SNI mice have a similar distribution of AM and DH fibers as sham and naïve animals (p>0.05). **C)** There was an overall difference in the distribution of Aβ fibers among SNI, sham and naïve mice (*p<0.05). There was a decrease in the proportion of slowly adapting (SA) Abeta fibers in SNI mice compared to sham (*p<0.5), although no difference was observed between SNI and naive animals (p>0.5). Distribution data was analyzed using a Chi-square analysis and Fisher’s exact *post hoc* tests.

**Figure 4:**
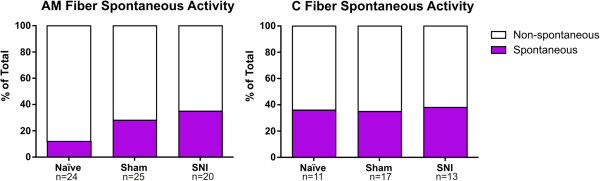
**Spontaneous activity is similar in AM and C fibers from naïve, sham, and SNI mice.** SNI mice did not differ from sham or naïve mice in that the percent of non-evoked spontaneous discharges was similar across treatment groups in either AM or C fibers (p>0.05). Data was analyzed using Chi-square analysis.

Sensory afferent sensitization is known to contribute to mechanical hypersensitivity observed in diabetic and chemotherapy-induced neuropathies [15,16], and has been shown to contribute to hypersensitivity observed in other models of nerve injury [10-12]. To assess the contribution of cutaneous sensory afferents to SNI-induced mechanical hypersensitivity, we performed *ex vivo* teased fiber recordings on the spared sural nerve. Fibers from SNI animals exhibited enhanced suprathreshold firing compared to controls. Specifically, Aδ-mechanoreceptor (AM) fibers fired an average of 22% more action potentials across all forces compared to sham or naïve mice, and C fibers exhibited 24% more action potentials across all forces compared to sham and 28% more than naïve mice (AM: Figure 2A, p < 0.01; C: Figure 2C, p < 0.05). Post hoc comparison showed no differences at individual forces for either AM or C fibers (Figure 2). There were no differences in mechanical thresholds or conduction velocity of any fiber subtype across treatment groups (Table 1). There was no difference in the percentage of Aβ, Aδ, and C fibers encountered in preparations from the different surgical groups (Figure 3A, p > 0.05). There was also no difference in the proportion of slowly adapting (AM) and rapidly adapting (D-hair) Aδ fibers (Figure 3B, p > 0.05). There was an overall difference in the distribution of Aβ fibers among SNI, sham and naïve mice (Figure 3C, p < 0.05). There was a decrease in the distribution of slowly adapting (SA) A-beta fibers from SNI animals compared to sham (Figure 3C, p< 0.5), although no difference was observed between SNI and naive animals (Figure 3C, p> 0.5). We also measured spontaneous activity because spontaneous activity in primary afferent fibers accompanies other nerve injury animal models including SNL and CCI [10-13,17-19]. There was no difference in the percentage of AM or C fibers that exhibited spontaneous activity in SNI versus sham or naïve groups (Figure 4, p > 0.05). Furthermore, preliminary analysis of Aβ fibers from SNI preparations also does not suggest increased spontaneous firing, as has been observed in SNL or CCI (data not shown). There was also no difference in the frequency of spontaneous action potential firing in SNI versus controls for any fiber type (data not shown). In dividing groups into early and late stages post SNI, there was no significant difference in the spontaneous activity of fibers recorded at POD 16–21 compared to those at POD 37–42 for Aβ, Aδ, or C fibers (data not shown).

**Table 1:** Summary of fiber properties in Naive, Sham and SNI mice

**Fiber type**	**Genotype**	**n**	**Median von frey threshold (mN)**	**Lower quartile**	**Upper quartile**	**Mean conduction velocity (m/s)**	**±SEM**
	Naive	24	6.82	6.82	13.88	4.30	0.41
Aδ-Mechanoreceptor	Sham	25	6.82	5.41	11.70	5.33	0.62
	SNI	20	6.82	4.00	11.70	3.95	0.71
	Naive	5	0.66	0.66	1.15	4.80	0.86
D-hair	Sham	7	0.66	0.27	0.66	4.93	0.80
	SNI	7	0.27	0.23	1.63	5.57	0.54
	Naive	11	6.82	6.82	11.70	0.66	0.10
C	Sham	17	11.70	5.41	14.60	0.68	0.06
	SNI	13	6.82	4.00	11.70	0.65	0.08
	Naive	7	1.63	0.66	4.00	11.60	1.05
RA-Aβ	Sham	10	0.66	0.66	1.63	13.99	1.38
	SNI	17	1.63	0.66	1.63	12.85	0.58
	Naive	8	1.63	1.63	3.41	13.47	1.46
SA-Aβ	Sham	13	1.63	.63	2.81	14.49	1.33
	SNI	11	1.63	0.66	4.00	14.76	1.15

To our knowledge, this is the first study to assess sensitization of primary afferent fibers in SNI. Our results suggest that enhanced suprathreshold firing in AM and C fibers may contribute to the robust behavioral mechanical hypersensitivity that occurs in the SNI model of neuropathic pain. Sensitized nociceptors might contribute to SNI-induced behavioral hypersensitivity either directly through increased suprathreshold firing in response to external stimuli, or indirectly by driving central sensitization [10,11]. Previous nerve injury studies that used the SNL and CCI models of nerve injury suggest that Wallerian Degeneration of injured nerves drives sensitization of adjacent intact afferent fibers [10-12]. However, unlike CCI and SNL, Wallerian Degeneration is not a major factor in the SNI model of neuropathic pain as SNI involves minimal co-mingling of intact and injured afferent fibers [1]. Therefore, a different mechanism(s) likely drives the afferent mechanical sensitization observed in this model. One potential mechanism is paracrine signaling between injured and intact cell bodies within the dorsal root ganglia (DRG), a level where co-mingling occurs. A previous study has shown an increase in macrophage infiltration, expression of inflammatory mediators such as IL-6 and TNF-α, and expression of neurotrophins BDNF and NGF in the DRG after sciatic nerve injury [20], and these may be key factors driving afferent sensitization in the SNI model [1]. Alternatively or in addition, at the peripheral terminals, collateral sprouting of intact sensory afferent terminals into the denervated skin territory of the transected nerves has been shown in other neuropathic pain models [21,22], and may also occur and contribute to sensitization in SNI.

It has been shown that degeneration of injured fibers induces spontaneous activity in nearby uninjured primary afferent fibers [23]. Furthermore, previous studies have shown that increased spontaneous activity in A and C fibers can contribute to sensitization after nerve injury in other models of neuropathic pain [10-13,17-19]. However, we did not observe more spontaneous activity in either A or C fibers post-SNI. One explanation for the absence of spontaneous activity may be that ectopic discharge rates change over time after injury. Previous studies on injured primary afferent fibers show that there is a higher frequency of spontaneous activity early after nerve injury (POD 1–3) and less activity in late stages (POD 11–14) [17,18]. Furthermore, studies on uninjured afferents, which show spontaneous activity, have been performed primarily at early stages after nerve injury [13,23]. Thus, our recordings at later stages (POD 16–42) after injury may have occurred after SNI-induced spontaneous activity subsided. Another likely explanation is that spontaneous activity may not be present in the SNI model due to minimal co-mingling of injured and adjacent fibers. Previous reports of spontaneous activity after nerve injury have been recorded from nerve injury models that involve considerable Wallerian Degeneration and extensive co-mingling of intact and injured axons [10-12]. Sensitizing compounds associated with Wallerian Degeneration, such as TNF-α, have been shown to sensitize primary afferent fibers [24]. However, in the absence of co-mingling of intact and injured axons distal to the site of lesion, and the minimal degeneration of injured fibers proximal to the lesion, these compounds may not affect the intact peripheral afferent fibers in the SNI model.

## Conclusions

These results may provide insight into the mechanisms underlying neuropathic pain in humans with traumatic peripheral nerve injury. Our results show an increase in suprathreshold firing in Aδ-mechanoreceptor (AM) and C fibers, suggesting that enhanced primary afferent drive may contribute to nerve injury-induced hypersensitivity, and peripheral afferent fibers may be targets for pharmacological treatment of neuropathic pain.

## Materials and methods

### Animals

Male C57BL/6 mice (Jackson Labs), 8 weeks at time of injury, were used for all behavioral and teased fiber skin nerve experiments. Animals were housed individually after surgery and handled equally during all experiments. All experimental protocols were approved by the Medical College of Wisconsin’s Institutional Animal Care and Use Committee.

### Surgery

SNI surgery was performed as previously described [1]. Briefly, under ketamine-induced anesthesia, animals underwent surgery to ligate and transect the left tibial and common peroneal nerves, and 2-4 mm of nerve distal to the ligation was removed to prevent regeneration. Care was taken to avoid damage to the sural nerve. Sham animals underwent anesthesia and skin and muscle incisions identical to the SNI animals, without ligation or axotomy of the tibial and peroneal nerves. Naïve animals received no surgical treatment or anesthetic. Although we attempted to blind the experimenter to surgery type, it was possible to distinguish which animals had undergone SNI, sham or naïve treatment.

### Behavior

Behavioral sensitivity to dynamic light touch was assessed on the tibial skin territory of the left paw as previously reported [14]. Mechanical threshold and sensitivity to suprathreshold mechanical force were assessed on the sural territory of the left hind paw as previously reported [14]. The animals were tested twice before surgery and on post-operative-days (POD) 1, 2, 4, 7, 14, 21, 28, 35, 42, 49, 90, and 180.

### Teased fiber skin-nerve recordings

Teased fiber recordings were used to determine the mechanical response properties of cutaneous primary afferent fibers in skin nerve preparations from SNI, sham and naïve mice as previously described [25]. Briefly, the sural nerve and innervated skin of the left hindlimb were dissected and placed corium side up in a recording bath superfused with oxygenated synthetic interstitial fluid at 32 ± 0.5°C. The nerve was desheathed and fascicles were teased apart until single, functionally distinct fibers, could be distinguished. Fibers were characterized by mechanical threshold and conduction velocity. Units with conduction velocities over 10 m/s were classified as Aβ, and Aδ for units with conduction velocities between 1.2 m/s and 10 m/s. C fibers were classified as units that had conduction velocities less than 1.2 m/s. Units were further sub-classified as slowly adapting (SA) or rapidly adapting (RA) based on the rate of adaptation to mechanical force. Following characterization, fibers were recorded for 2 min to assess spontaneous activity. Next a feedback-controlled mechanical stimulator was used to deliver increasing sustained mechanical forces (5-200 mN) for 10 sec each with 1 min recovery period between stimuli. Action potentials were recorded and analyzed using Lab Chart Data Acquisition Software (AD Instruments, Colorado Springs, CO).

### Data analysis

All data sets were compared between SNI, sham, and naïve groups. Behavioral Data: Percent response to light touch was analyzed across time using repeated measures two-way ANOVA with Tukey’s *post hoc* test. Mechanical withdrawal thresholds and the percent response to a 3.31 mN monofilament were compared across time (Baseline 1 through POD 49) using a 2-way ANOVA with Bonferroni *post hoc* analysis. Withdrawal thresholds for SNI and sham were compared POD 90 and POD 180 with Mann Whitney U tests. Skin-Nerve Data: Each fiber type was analyzed for: 1) number of action potentials fired across mechanical forces using a two-way ANOVA with Bonferroni *post hoc* comparisons, 2) conduction velocity using a one-way ANOVA with Tukey’s multiple comparisons, 3) von Frey thresholds using Kruskal-Wallis with Dunn’s multiple comparisons, and 4) percent spontaneous fibers using Chi-square analysis. Column statistics of each fiber type were analyzed to compare the sum of the average number of action potentials fired across all forces. Percent distribution of the fiber types was compared using Chi-square analysis and Fisher’s exact *post hoc* tests. Data analysis was completed using Prism 6 Software (GraphPad, La Jolla, CA).

## Abbreviations

SNI: Spared nerve injury; CCI: Chronic constriction injury; SNL: Spinal nerve ligation; POD: Post, operative day; AM: Aδ-Mechanoreceptor; DRG: Dorsal root ganglia.

## Competing interests

The authors declare that they have no competing interests.

## Authors’ contributions

All authors read and approved the final manuscript. AS and CO conducted the experiments and analyzed the data. AS and CS designed the study and wrote the manuscript.
